# The Arctic Daisy, *Chrysanthemum arcticum*, Trifecta Is a Genetically Variable Polyploid Series

**DOI:** 10.3390/genes17040444

**Published:** 2026-04-13

**Authors:** Liesl Bower-Jernigan Litschewski, Neil O. Anderson, Laura M. Shannon

**Affiliations:** Department of Horticultural Science, University of Minnesota, Saint Paul, MN 55108, USA; llitschew001@csbsju.edu (L.B.-J.L.); lmshannon@umn.edu (L.M.S.)

**Keywords:** ploidy variation, flow cytometry, aneuploidy, polyploidy, *Chrysanthemum arcticum* subsp. *arcticum*, *Chrysanthemum arcticum* subsp. *polaré*

## Abstract

Background/Objectives: Accurate ploidy determination is essential for understanding population structure and evolutionary history for breeding and domestication. The *Chrysanthemum arcticum* (Arctic daisy) complex, comprising *C. arcticum* and subspecies *arcticum* and *polaré*, exhibits variability in ploidy variation with reports ranging from diploid (2*n* = 2*x* = 18) to octoploid (2*n* = 8*x* = 72). Methods: The objective of this study was to assess ploidy levels in *n* = 225 genotypes from *n* = 46 wild, native populations of the three taxa collected across mainland Alaska, Attu Island (Alaska), and Hudson Bay (Canada) using flow cytometry, and using *C. nankingense* (2*n* = 2*x* = 18) as the diploid reference. Results: Genome sizes were from 5.27 to 20.69 pg (2C), corresponding to diploid through hexaploid. Triploids were the most frequent (64%) before, as well as after, applying the reference standard bias (10% correction). All ploidy levels were present in multiple geographic regions, with no clear spatial, taxonomic, or latitudinal segregation. The high incidence of triploids, most of which were self-compatible and highly fertile, may reflect genetic instability, underlying aneuploidy (which is common in several *Chrysanthemum* polyploids), or systematic bias from reference standard differences. Inconsistencies between flow cytometry estimates and observed reproductive compatibility, such as successful crossings with known diploids, suggest additional genomic complexity. Potential historical influences creating genomic instability, including environmental disturbances (chemical, radiation warfare remnants) from World War II military activities at Attu Island and Hudson Bay, are discussed. Conclusions: This study shows the challenges of accurately determining ploidy levels in the *C. arcticum* trifecta complex and highlights the need for other approaches, including high-density SNP genotyping and chromosome imaging, to resolve ploidy questions and guide future breeding strategies.

## 1. Introduction

The genus *Chrysanthemum* L. (Asteraceae) comprises *n* = 75 [1] or more [2] species, depending on whether the species in the *Chrysanthemum* complex are lumped or split by taxonomists. These species are traditionally classified into the following three sections: *Chrysanthemum*, *Ajania* Poljakov, and *Arctanthemum* Tzvelev. Taxonomic revisions have gone back and forth between the names *Dendranthema* (DC.) Des Moul. and *Chrysanthemum* [2,3,4,5,6,7]. All species in the *Chrysanthemum* complex share a base chromosome number (ploidy) of *x* = 9 [8,9,10,11].

While *Chrysanthemum* is well characterized for many cultivated and wild taxa, reports for *C. arcticum* are inconsistent. *Chrysanthemum arcticum* L. has been identified as diploid (2*n* = 2*x* = 18) [12] and octoploid (2*n* = 8*x* = 72) [13] using root tip squash preparations. Resolving these discrepancies is important for understanding the species’ evolutionary history, adaptive strategies, and potential role in breeding programs.

Cultivated chrysanthemums (*Chrysanthemum* × *grandiflorum* Tzvelv., *C.* × *hybridum* Anderson) are the most widely grown taxa [14], which are all allohexaploid (2*n* = 6*x* = 54), exhibiting somatic chromosome numbers ranging from 2*n* = 47 to 63 due to aneuploidy [15]. The gain/loss of chromosomes in clonal propagation of cultivars can change the flower color [16,17]. The species is characterized by self-incompatibility (SI), controlled by ≥2 *S* loci [18], but can display pseudo-self-compatibility (PSC) under certain conditions [19]. Breeding is further complicated by frequent aneuploidy [17], reduced fertility and/or sterility [20], inbreeding depression [21], and high genetic load [22]. These factors collectively present significant challenges for the development of new cultivars in polyploid chrysanthemum taxa.

*Chrysanthemum nankingense* (Nakai) Tzvelv., a diploid species (2*n* = 2*x* = 18), has long been considered a potential progenitor of cultivated chrysanthemums. Genomic studies reveal that *C. nankingense* underwent repetitive element expansion and a whole-genome duplication event [23,24]. However, recent chromosome-scale genome analyses indicate that the domestication of cultivated chrysanthemums likely involved multiple wild diploid and polyploid species, and *C. nankingense* may not be the sole or direct ancestral donor [25]. *Chrysanthemum nankingense* was selected as the diploid standard due to its close phylogenetic relationship to *C. arcticum*, a sequenced genome, and germplasm availability.

Characterizing ploidy levels is necessary for understanding population structure and evolution in a species, particularly with their impact on breeding and crop domestication [26]. The morphology, anatomy, physiology, and biochemistry of an organism are affected by its number of chromosomes. There is also a correlation between ploidy level and the mode of reproduction [26]. Many polyploid chrysanthemum taxa are self-incompatible while diploids are either self-compatible or self-incompatible. A thorough investigation is required to determine the ploidy within and across the *Chrysanthemum arcticum* complex. The objective of this study is to determine the ploidy levels of all *C. arcticum* species complex germplasm collected in the wild (extant populations) and maintained at the University of Minnesota to determine the differences in ploidy between species and subspecies.

## 2. Materials and Methods

### 2.1. Germplasm

#### 2.1.1. Clonal

This study focused on the exact extant wild population collections by Dr. Neil Anderson (University of Minnesota) that have been studied for phenomics and genomics [27,28,29]. In 2017 and 2018, rhizome samples of 225 genotypes of extant *C. arcticum* from nine populations and 326 genotypes of extant *C. a.* subsp. *arcticum* from 21 populations were collected [28]. In the summer of 2019, 375 genotypes of extant *C. a.* subsp. *polaré* were collected from 20 populations [27,28,29]. Rhizomes were transplanted into a mist house equipped with an intermittent mist system (10 min intervals; reverse osmosis water) for rooting. After 1–2 weeks of rooting, plants were transferred to an environmentally controlled glass greenhouse maintained at 24.4 ± 3.0 °C (day) and 18.3 ± 1.5 °C (night) under a 16 h photoperiod (0600–2200 HR; long days). During winter, supplemental lighting was provided by 400 W high-pressure sodium high-intensity discharge (HPS-HID) lamps, maintaining a minimum of 150 μmol m^−2^ s^−1^ at plant level. The greenhouse, located at the University of Minnesota’s St. Paul campus Plant Growth Facilities, was managed by a computerized environmental control system. Plants received fertigation twice a day, between 0700 and 0800 HR and 1600–1700 HR, with a constant liquid feed (CLF) delivering 125 ppm N from a water-soluble 20N–4.4P–16.6K fertilizer (Scotts, Marysville, OH). Monthly rotational fungicide drenches were also applied. Each year all the plants were given a 6-week cold treatment (3–5 °C, walk-in cooler).

Seeds of *C. a.* subsp. *polaré* accessions collected in Nome, AK, were obtained from the U.S. Department of Agriculture Germplasm Resources Information Network (USDA-GRIN, W6 54572, *Chrysanthemum arcticum* L. subsp. *polaré* Hultén, AK930-790) and grown under the same conditions (*n* = 85). *Chrysanthemum nankingense* (Nakai) Tzvelv. (2*n* = 2*x* = 18) was obtained from Etsy (https://www.etsy.com/market/chrysanthemum_nankingense (accessed on 12 March 2024)) and used as the diploid comparison [30] and grown in the same greenhouse as all the other samples. Genotypes were sampled individually for *C. arcticum* and pooled for *C. a.* subsp. *arcticum* and *C. a.* subsp. *polaré* (Table 1) as per pooling methodology of [31].

#### 2.1.2. Seedlings

Select clonal parents of *C. arcticum* were used to create F_1_ hybrid and segregating populations for heterogeneity tests. The crosses performed among parents and their progeny numbers (20Ca-69, 20Ca-71, 20Ca-72, 20Ca-73, and 20Ca-75) are in Table 2. All parents were assumed to possess PSC [19], rather than self-incompatibility [18], and were emasculated prior to cross pollination, using standard hybridization techniques used in the genus [18,20]. Since each *Chrysanthemum* flower is an inflorescence [18,19,20], containing hundreds of ovules in ray (gynoecious) and disk (hermaphroditic) florets, only *n* = 1 flower was needed to generate enough seeds. The number of pollinations (inflorescences), total seed set (number of seeds sown), and number (%) germinated seedlings used in the experiment are shown in Table 2.

### 2.2. Flow Cytometry Methodology

Flow cytometric analysis of *C. arcticum* nuclei was performed using the CyStain^®^ PI Absolute P kit (Partec GmbH, Jettingen-Scheppach, Germany; product no. 05-5022), following the manufacturer’s protocol for nuclei extraction and PI (propidium iodide) staining. The most recently fully expanded leaves below the apical meristem were sampled from each plant. For each genotype, *n* = 3 samples were taken from the same plant using different shoots.

During analyses, if any bulked population showed multiple peaks due to genotypic variation, the genotypes were separated and analyzed individually (Table 1). Thus, the bulked samples had cytogenetic uniformity and were not analyzed separately (Table 1).

### 2.3. Sample Analyses

A BD Accuri™ C6 Plus Flow Cytometer (BD Accuri™ C6 Plus Flow Cytometer, BD BioSciences, San Jose, CA, USA) was used to collect data, and the associated program (BD Accuri C6 Plus software and BD CSampler Plus software, BD BioSciences, San Jose, CA, USA) was used to analyze the samples. *Chrysanthemum nankingense* was used as diploid comparison and samples were run every day. The mean PE-A (phycoerythrin-A) for all samples and histograms was recorded. PE-A was used because the excitation level of PI is within the range of PE. Phycoerythrin is excited at a maximum of 565 nm and emits at 573 nm.

The mean fluorescence intensity (mean PE-A) was recorded for each replicate sample and for each of the bulked samples. For each genotype, the average and standard deviation of mean PE-A values were calculated. DNA content was estimated following the approach of Qiu et al. [32] for Equation (1):

(1) $$Sample\;2C\;DNA\;content=\;\frac{Sample\;G1\;peak\;mean}{Standard\;G1\;peak\;mean}\;\times \;Standard\;2C\;DNA\;content\;(pg\;DNA)$$

For calibration, the mean of mean PE-A values for *C. nankingense* was determined and used as the internal standard (2C = 6.63 pg DNA). The mean of each sample’s mean PE-A was divided by the standard’s mean and multiplied by 6.63 pg to obtain the estimated DNA content. Standard deviations were calculated for all genotypes to assess the variation within populations.

### 2.4. Heterozygosity Analysis

We retained five crosses for which both parents and their offspring had SNP genotype data. For each cross we identified markers which were segregating with at least one parent and had offspring genotype calls. Offspring genotypes which contained alleles that were not present in the parent were set to missing; markers with more than three inappropriate offspring genotype calls were removed. We classified markers into two classes—one parent segregating or both parents segregating. For each marker class in each parent, we counted offspring genotypes. These counts were compared to expected values using a Chi-squared test.

For diploids, the expected ratio for genotypes with a single parent segregating is 50% heterozygous: 50% homozygous (class I is the sum of the DArTseq SNP genotype calls 1 × 0 and 2 × 1) [27,28]. When two parents are segregating the expected ratio is 50% heterozygous: 25% of each homozygous class (class II is the sum of 1 × 1). In autotetraploids, the three heterozygous classes (Aaaa, AAaa, and AAAa) would be pooled into a single class, based on genotyping technology. The first class of markers could, therefore, be an aaaa × aaaA cross, an aaaa × aaAA cross, or an aaaa × aAAA cross. The first of these possibilities would result in 50% homozygous: 50% heterozygous offspring. The second case would result in 1/6 (16.7%) homozygous: 5/6 (83.3%) heterozygous individuals. The third case would result in 100% heterozygous individuals. Using the DArTseq SNP data all three of these crosses would appear to be aa × aA and we could not distinguish between them. If we take the average of the three possibilities, we arrive at an expected value of 78% heterozygosity and 22% homozygosity. For markers with two segregating parents there are nine possible crosses, all of which would appear as aA × aA. Crosses of type simplex (aaaA) × simplex produce 25% homozygous: 75% heterozygous offspring. Simplex × duplex (aaAA) crosses produce 1/12 (8.3%) homozygous and 11/12 (91.7%) heterozygous offspring, as would duplex × triplex (aAAA) crosses. All offspring of simplex × triplex crosses are heterozygous. Duplex × duplex crosses produce 1/36 (2.8%) offspring of each homozygous class and 34/36 (94.4%) heterozygous offspring. Triplex × triplex crosses are identical to simplex × simplex. If we assume all genotypes are equally likely, the expected heterozygosity of the offspring is 90%, while we would expect 5% of each class of homozygous offspring.

For triploids individuals which appear as aA in the genotyping data may be aAA or aaA. Sexual reproduction is rare in triploids, but if it occurs, gametes may be either monoploid or diploid. Again, this is impossible to distinguish so we rely on an average value. For an aAA × aaa cross, the expected homozygosity is 1/6 (16.7%) and the expected heterozygosity is 5/6 (83.3%). For an indistinguishable aaA × aaa cross, the expected heterozygosity and homozygosity are both ½ (50%). Then, for an Aa × aa cross, the average expected homozygosity is 1/3 (33.3%) and the average expected heterozygosity is 2/3 (76.7%). For an aAA × aAA cross, the expected heterozygosity is 13/18 (72.2%), the expected homozygosity is 1/36 (2.8%) and the expected A homozygosity is 9/36 (25%). For an aAA × aaA cross, the expected heterozygosity is 8/9 (88.9%) and the expected homozygosity for each allele is 1/18 (5.6%). Thus, for an apparent aA × aA cross, if the underlying genotypes are triploid, the expected heterozygosity is 81% and the expected homozygosity is 9.5% for each allele.

## 3. Results

### 3.1. DNA Content Measurement

**Figure 1:**
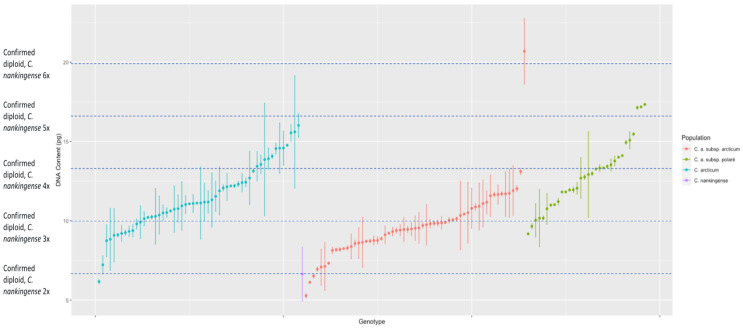
Estimated DNA content (2C) for *Chrysanthemum arcticum* complex genotypes or bulked homogeneous samples grouped by species. Genome sizes were determined via flow cytometry using *C. nankingense* as the diploid reference standard. Data represent measurements for 225 genotypes.

**Figure 2:**
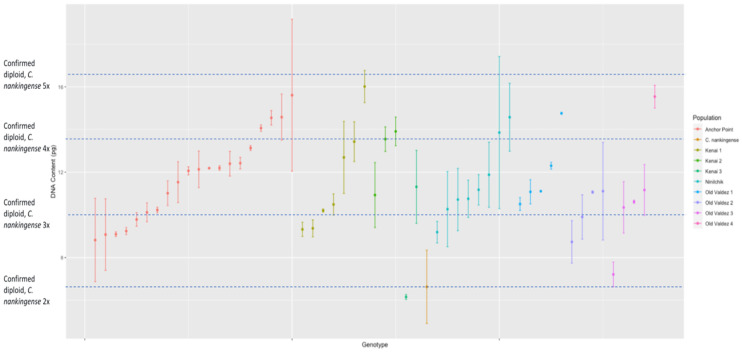
Estimated DNA content (2C) for *Chrysanthemum arcticum* grouped by population and genotype. Genome sizes were determined via flow cytometry using *C. nankingense* as the diploid standard reference.

Flow cytometry analysis of the *n* = 225 *Chrysanthemum arcticum* complex genotypes revealed substantial variation in genome size (Figure 1). The smallest genome measured was 5.27 pg (2C) in *C. a.* subsp. *arcticum* (Attu-1–17), while the largest was 20.69 pg (2C) in the same subspecies but a different population (Attu-13–6; Figure 1). Across all genotypes, DNA content values were consistent within genotypes, as indicated by the low within-genotype standard deviations (mean = 0.62 pg; range = 0.01–3.56 pg). Using *C. nankingense* as the diploid reference standard (2*n* = 2*x* = 18), there is a range of ploidy within the *C. arcticum* complex (Figure 2).

**Figure 3:**
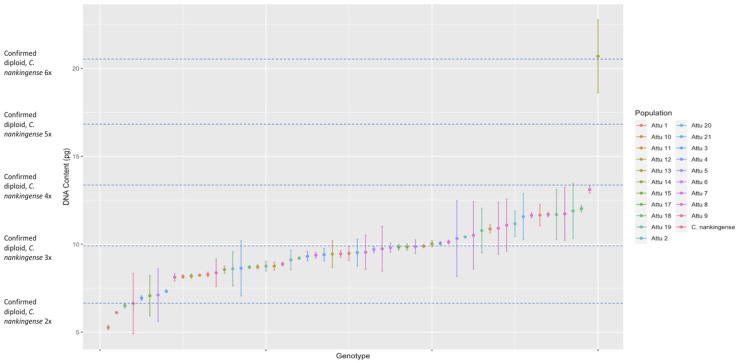
Estimated DNA content (2C) for *Chrysanthemum arcticum* subsp. *arcticum* genotypes or bulked homogeneous samples grouped by population. Genome sizes were determined via flow cytometry using *C. nankingense* as the diploid standard.

**Figure 4:**
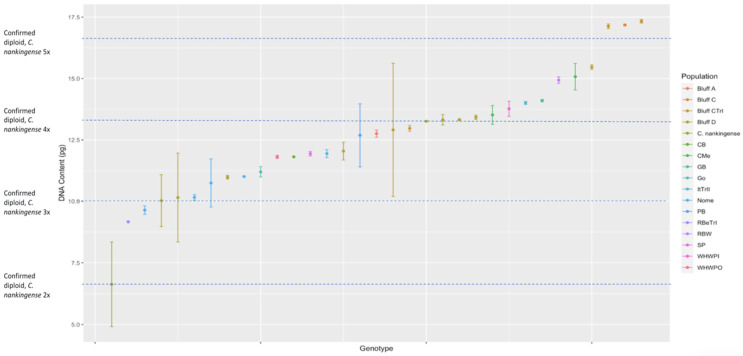
Estimated DNA content (2C) for *Chrysanthemum arcticum* subsp. *polaré* genotypes or bulked homogeneous samples grouped by species. Genome sizes were determined via flow cytometry using *C. nankingense* as the diploid standard.

The DNA content measured from the *C. arcticum* complex shows a continuous distribution for all taxa rather than having clear segregation for ploidy levels. This hampered our ability to categorize exact ploidy levels with the added confounding of triploid occurring in fertile individuals. Instead, we categorized the distribution of genotypes within mean DNA content based on the diploid *C. nankingense*: 2× = DNA content, 3× = DNA content × 1.5, 4× = DNA content × 2, 5× = DNA content × 2.5, and 6× = DNA content ×3 (all taxa, Figure 1; *C. arcticum*, Figure 2; *C. a.* subsp. *arcticum*, Figure 3; and *C. a.* subsp. *polaré*, Figure 4).

Within all taxa, four genotypes (1.8%) had DNA contents below that of the known diploid (Figure 1). Forty-eight genotypes (21.3%) fell between the diploid comparison DNA content and ×1.5 of diploid comparison. Sixty-six genotypes (29.3%) fell between the diploid comparison ×1.5 and ×2 of diploid comparison. Twenty-three genotypes (10.2%) fell between the diploid comparison ×2 and ×2.5 of diploid comparison (Figure 1). Three genotypes (1.3%) fell between the diploid comparison ×2.5 and ×3 of diploid comparison. One genotype (0.4%) fell between the diploid comparison ×3 and ×3.5 of diploid comparison.

Analyses of the *C. arcticum* populations Anchor Point, Kenai 1, Kenai 2, Kenai 3, Ninilchik, and Old Valdez 1 showed a larger range in distribution of DNA content (Figure 2). Populations Old Valdez 2, Old Valdez 3, and Old Valdez 4 have a smaller range in distribution of DNA content (Figure 2).

The populations within *C. a.* subsp. *arcticum* fell under ×1 diploid standard, between ×1 diploid standard and ×1.5 diploid standard, and between ×1.5 diploid standard and ×2 diploid standard, with the one exception of one genotype within Attu 13 falling above ×3 diploid standard (Figure 3).

**Figure 5:**
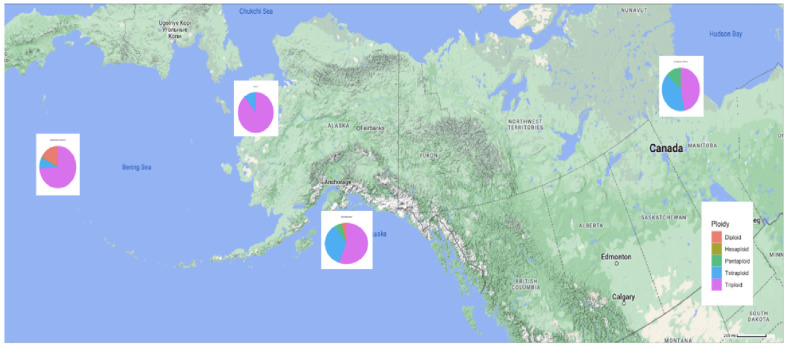
Geographic distribution of ploidy levels by collection site for the *Chrysanthemum arcticum* complex [27,28,29]. Each site’s pie chart indicates the relative proportion of diploid, triploid, tetraploid, pentaploid, and hexaploid genotypes.

The populations of *C. a.* subsp. *polaré* have a widespread distribution with the populations falling between ×1 diploid standard and ×3 diploid standard (Figure 4). Most genotypes fell between ×1.5 diploid standard and ×2 diploid standard and ×2 diploid standard and ×2.5 diploid standard (Figure 4). There is no clear geographic separation of ploidy levels within or among the three taxa in the Arctic daisy complex (Figure 5).

### 3.2. Heterozygosity

Using a Chi-squared test with *n* = 4 degrees freedom (df; number of crosses −1), the observed segregation ratios did not fit any of the models (Table 3). For heterozygous parent by homozygous alternate allele parent markers, the observed heterozygosity ratio was higher than any of the three expected heterozygosity ratios in all families (Table 3). For the heterozygous parent by homozygous reference allele parent markers, the observed heterozygosity ratio was lower than that expected in any model (Table 3), indicating reference bias. The observed heterozygosity ratio for heterozygous by heterozygous markers in every family was between 71% and 75% (Table 3), too high for the expected diploid ratio and too low for the expected triploid ratio.

**Figure 6:**
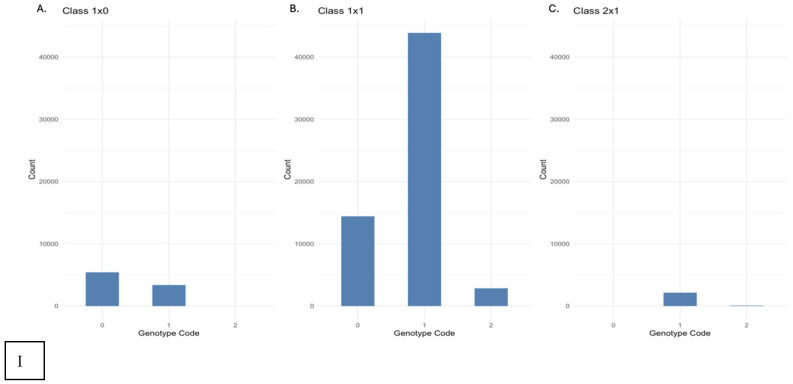

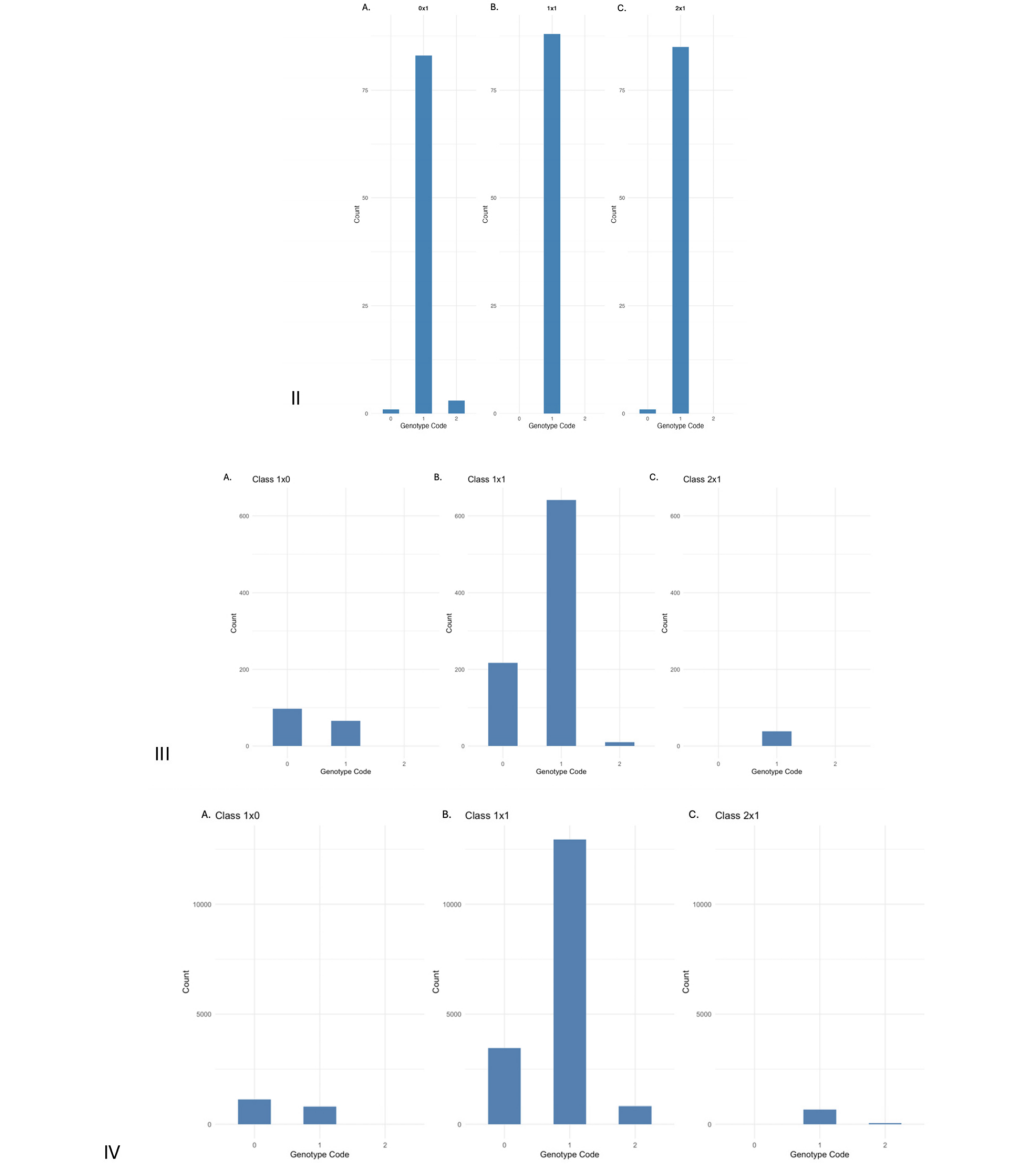

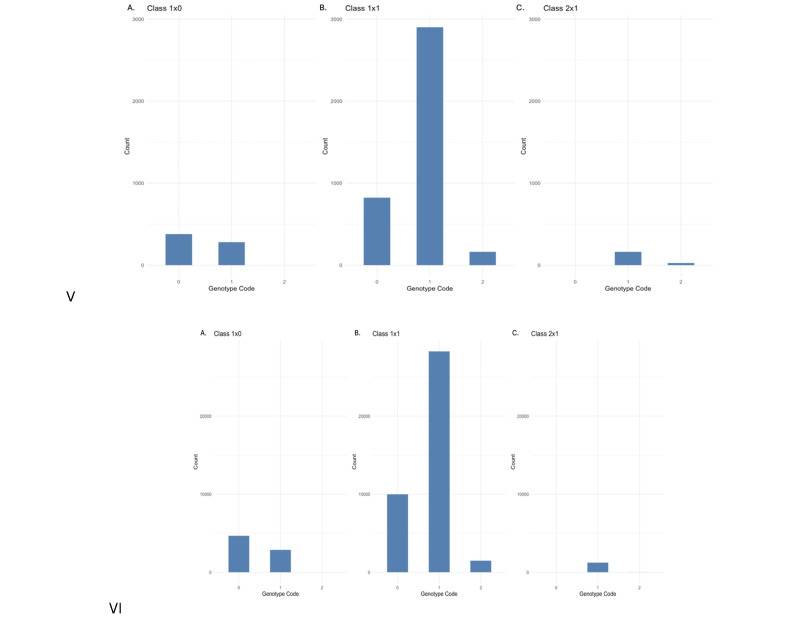
Distribution of genotype codes in classes from DArTseq SNP data [27,29]: [**I**] (A) Class 1 × 0; (B) Class 1 × 1; (C) Class 2 × 1 within cross 20Ca-75; [**II**] for specific markers within cross 20Ca-75, (A) Class 1 × 0 (35811885|F|0–45:A>G-45:A>G); (B) Class 1 × 1 (66756243|F|0–20:A>G-20:A>G); (C) Class 2 × 1 (35811164|F|0–34:G>A-34:G>A); [**III**] (A) Class 1 × 0; (B) Class 1 × 1; (C) Class 2 × 1 within cross 20Ca-69; [**IV**] (A) Class 1 × 0; (B) Class 1 × 1; (C) Class 2 × 1 within cross 20Ca-71; [**V**] (A) Class 1 × 0; (B) Class 1 × 1; (C) Class 2 × 1 within cross 20Ca-72; [**VI**] (A) Class 1 × 0; (B) Class 1 × 1; (C) Class 2 × 1 within cross 20Ca-73.

In testing the cross with the most genotypes (Figure 6I), the distribution of genotypes does not display a diploid, triploid, and/or tetraploid pattern. When looking at markers within 20Ca-75 (Table 2) for each class, there are very high levels of heterozygosity for each class (Figure 6II). All other distribution patterns for tested crosses showed varying results within crosses 20Ca-69 (Figure 6III), 20Ca-71 (Figure 6IV), 20Ca-72 (Figure 6V), and 20Ca-73 (Figure 6VI) for genotype codes from DArTseq SNP data [27,29] in classes 1 × 0, 1 × 1, and 2 × 1.

## 4. Discussion

Previous reports identified the ploidy levels of *C. arcticum* as two distinct groups, diploid [12] and octoploid [13]. This study shows a range of DNA content exceeding expectations for a diploid (Figure 5). We attempted karyotyping to determine actual chromosome counts. This was unsuccessful since no cells could be found at the proper stage of mitotic division to visualize the small chromosomes characteristic of *Chrysanthemum* taxa. However, flow cytometry and karyoptic analyses are comparable in chrysanthemum [33]. For example, flow cytometry identified *Chrysanthemum* × *grandiflorum* Tzvelv. cultivars to be triploid, tetraploid, pentaploid, hexaploid to heptaploid, like karyotypic analysis [33]. Similarly, the range of ploidy in *C. arcticum* mirrors that of *C.* × *grandiflorum* in having a high percentage of triploids. Due to large genome size and high heterozygosity, *Chrysanthemum arcticum* is not a diploid but rather a highly variable polyploid series. With few exceptions (e.g., Attu 13, Figure 3), there is a continuous distribution of DNA content throughout the complex (Figure 1). However, the expectation would be clear ploidy designations with gaps between ploidies as in *Triticum aestivum* L. [34]. This was not the case across the Arctic daisy complex. Instead, Arctic daisy taxa are similar to *Festuca ovina* L., where a continuous distribution in DNA content occurs between tetraploid and hexaploid genotypes [35], and the ancient polyploid, *Arachis hypogea* L. (2*n*  =  4*x*  =  40), with genetic and chromosomal instability [36,37,38].

If ploidy was assigned, many of the Arctic daisy genotypes would be classified as triploids (Figure 1). Despite these estimates, the ploidy assignments presented notable inconsistencies when compared with physiological and reproductive observations. Many plants capable of crossing with known diploids were also self-compatible or PSC, rather than SI, with high levels of fertility and fecundity [39], a trait in chrysanthemums generally associated with diploids [40]. The implausibility that any triploids are fertile and self-compatible indicates that other factors that create the range of DNA content coexist within and among populations, without clear geographic or taxonomic partitioning (Figure 1 and Figure 5).

There is a range of ploidies within the genus *Chrysanthemum*. Several wild species are diploid (2*n* = 2*x* = 18), such as *C. seticuspe*, *C. coronarium*, *C. carinatum*, *C. segetum*, and *C. nankingense* (the standard reference used in this paper), and serve as important models for chrysanthemum genetics [24]. Tetraploids have also been reported. For example, *C. zawadskii* and *C. indicum* include cytotypes ranging from diploid to hexaploid (including tetraploids), depending on geographic origin and population history [41], aligning with the range of DNA content occurring in the *C. arcticum* complex (Figure 1 and Figure 5). Cultivated chrysanthemums (*C.* × *grandiflorum*) are allohexaploid (2*n* = 2*x* = 54) derived from >10 species, but are rarely stable, commonly exhibiting somatic aneuploidy, with reported chromosome numbers ranging from 2*n* = 2*x* = 47 to 63 [15,17]. These cytogenetic irregularities (gain or loss of a single chromosome) can result in significant phenotypic alterations (sports), such as changes in flower color, flower type, fertility, and/or growth habit [16]. Similarly, the genomic diversity of *Chrysanthemum* species shows that such variation is not restricted to whole-genome duplication but often includes aneuploidy, segmental duplications, and hybridization [25]. Aneuploidy irregularities of *C*. × *grandiflorum* generate a “boom” in phenotypic variation which has been useful in flower breeding to create a mutant series from a selected clonal cultivar, differing only by flower color [42]. For example, the “Charm” mutant family or series of plants, consisting of the cultivars “Coral Charm”, “Dark Bronze Charm”, “Dark Charm”, and “Salmon Charm”, are either spontaneous sports (somatic mutants) or radiation-induced sports of “Charm” [42]. These cultivars could not be genetically identified by DNA amplification fingerprinting (DAF) but were distinguishable with arbitrary signatures from amplification profiles (ASAPs). Forty-six (32%) unique amplification products were associated with the “Charm” cultivars with estimated mutation rates from 0.03% to 1.6% of nucleotide changes within 18 kb of an arbitrarily amplified DAF sequence.

This “boom” in phenotypically different mutants or sports due to nucleotide changes in arbitrarily amplified DAF sequences within hexaploid chrysanthemum is similar to recent discoveries in polyploid *Arachis hypogea* also displaying high frequencies of chromosomal or genetic instability [36,37,38]. The continuous range of DNA content observed in the *C. arcticum* polyploid complex parallels findings in other plant taxa where hybridization and genome size variation obscure discrete cytogenetic categories. In *Diphasiastrum* (Lycopodiaceae), genome size measurements revealed a continuous spectrum of cytotypes rather than sharply defined ploidy classes [43]. This pattern is attributed to frequent introgressive hybridization and a complex evolutionary history [43]. Similarly, our results show overlapping DNA content ranges across ×1 diploid standard through ×3 diploid standard, with no clear geographic or taxonomic separation (Table 3, Figure 6I). Thus, *C. arcticum* may also represent a complex genetic evolutionary history, where hybridization, aneuploidy, and potentially variation in base chromosome number contribute to the lack of distinct ploidy boundaries. Chromosomal instability may also explain the poor-quality SNP data and high rate of missing markers [27,29], which hindered efforts to estimate ploidy using SNP data.

Flow cytometry-based genome size estimates rely heavily on the accuracy of the chosen reference standard. If the plant standard used does not have the same DNA content per ploidy level as the study species, genome size calculations and, thus, ploidy estimates can be systematically biased. Recent work recalibrating common plant standards has shown that values can deviate by more than 10%, and in some cases by over 30%, depending on tissue type, buffer, and fluorochrome used [44]. However, incorporating a 10–30% deviation did not eliminate the continuous range of flow cytometric data. For *Chrysanthemum arcticum*, using *C. nankingense* as a diploid reference assumes that both species share similar DNA content per chromosome set even though they are native to different continents. If the genome of *C. arcticum* is larger, this could result in an overestimation of ploidy (e.g., a true diploid appearing as a triploid). As Drescher et al. [45] emphasized, flow cytometry is susceptible to methodological artifacts ranging from sample preparation and staining to gating and compensation errors.

Historical factors may have influenced genomic stability in some populations. Several sampling sites were locations of documented World War II military activity, including Attu Island, the site of the only land battle fought on U.S. soil [46], and Hudson Bay, which hosted multiple military installations [47]. The Battle of Attu (11–20 May 1943) brought large-scale environmental disturbance through the construction of airfields, fortifications, and related infrastructure, combined with bombardment and troop movements. Similarly, Hudson Bay military outposts altered landscapes through land clearing, building, and supply operations. At both locations, military hardware and warfare waste remains without any environmental cleanup. Many of the taxa and populations collected were in close proximity to the military installations. Environmental stressors from these wartime activities, including soil compaction, chemical contamination, altered hydrology, and the introduction of non-native plant material, can disrupt meiotic chromosome segregation and increase the likelihood of aneuploid formations [48,49]. The genomic variation observed may reflect not only natural evolutionary processes but also historical impacts.

Polyploidy within the *C. arcticum* complex is consistent with global patterns showing increased polyploid incidence in stressful environments. Across angiosperms, polyploid frequency rises with higher latitudes, with the Arctic flora having higher incidences of recently derived polyploids [50,51].

Harsh coastal environments offer another useful potential cause for aneuploidy and polyploidization. Polyploidization can promote survival under tidal fluctuation and salinity stress, as demonstrated by the emergence of polyploid lineages such as *Spartina anglica* in European salt marshes and with the coexistence of diploid and triploid cytotypes in the halophyte turfgrass *Paspalum vaginatum* [52,53,54]. All three *C. arcticum* taxa grow in high salt level conditions which could impact ploidy levels [28].

At the genomic level, changes in transposable element activity may also underlie shifts in genome size and obscure clear ploidy boundaries [55]. The expansion of repetitive elements, together with hybridization in the wild [56,57] and variation in chromosome size among populations [58], may underlie the DNA content ranges observed. These molecular factors, interacting with ecological pressures, could reinforce the cytogenetic instability across *C. arcticum* populations and help explain the flow cytometry anomalies detected in this study.

These causal factors may corroborate that the cytogenetic diversity observed in *C. arcticum* populations may be shaped not only by historical hybridization and aneuploidy but also by ecological selection that favors polyploid cytotypes in high-latitude, maritime environments. Environmental stressors such as severe coastal weather, high latitudes, and even historical disturbances like World War II military activity may have contributed to the genomic instability observed in the Arctic daisy populations. Ecological pressures alone may not fully explain the continuous ploidy variation detected.

The observed levels of heterozygosity are unexpected for a diploid model. The high heterozygosity (Table 3) could be due to reads mapping to multiple homeologs which could indicate *C. arcticum* is an allopolyploid. Heterozygosity levels could also be due to extreme environments related to the environmental stressors from wartime activities, higher latitude, and soil salinity. Due to the severe climate conditions in which all taxa occur, heterozygosity and/or polyploid series would be advantageous for wider adaptation and longevity in these environments.

It is unclear how the DArT analysis can distinguish differing homeologs. Genetic techniques, particularly high-quality SNP genotyping, could offer a way to detect complex ploidy variation provided that dense, high-quality SNP datasets are available [59,60,61]. We attempted this approach using available SNP data. However, due to low read depth and sparse marker density, along with poor-quality SNP calls and a high proportion of missing data, we were unable to extract reliable ploidy information. A higher-density, higher-quality SNP dataset might overcome these limitations. We also attempted karyotyping and were unsuccessful. The challenges that polyploids create for flow cytometric analysis would benefit from obtaining read depth as well as classic karyotyping.

## 5. Conclusions

Cumulatively, these results underscore both the complexity and the challenges of accurately determining ploidy in the *Chrysanthemum arcticum* complex. To resolve these uncertainties, future research should include using high-density single-nucleotide polymorphism (SNP) genotyping for read depth (which would clarify whether the taxa are auto- vs. allopolyploid) and karyological studies to determine actual ploidy levels and whether the base number differs in the *C. arcticum* complex.

## Figures and Tables

**Table 1:** Population site codes, latitudes and longitudes, and population sample size (n) for the nine *Chrysanthemum arcticum* (mainland Alaska), 20 *C. arcticum* subsp. *arcticum* (Attu Island) [29], and 16 *C. arcticum* subsp. *polaré* (Hudson Bay, Canada and Nome or Sitŋasuaq (Inupiaq), Alaska) [27] populations collected for the center of each population.

Taxa, Population Site Code	Latitude	Longitude	Population Size (*n*)	Bulked Sample (*n*)
*Chrysanthemum arcticum*				
Old Valdez-1	61°6′52.1964″ N	146°16′0.5550″ W	5	-
Old Valdez-2	61°6′52.7070″ N	146°16′1.3650″ W	4	-
Old Valdez-3	61°6′47.6418″ N	146°16′0.7284″ W	4	-
Old Valdez-4	61°7′59.7354″ N	146°17′42.4062″ W	1	-
Anchor Point-1	59°46′25.7484″ N	151°51′57.5748″ W	20	-
Ninilchik	60°1′45.1128″ N	151°42′13.4280″ W	8	-
Kenai-1	60°31′31.4898″ N	151°12′34.6782″ W	7	-
Kenai-2	60°31′31.6590″ N	151°12′35.7006″ W	3	-
Kenai-3	60°32′5.7192″ N	151°12′44.3880″ W	2	-
Total			54	-
*Chrysanthemum arcticum* subsp. *arcticum*				
Attu-1	52°48′41.7780″ N	173°18′2.8440″ E	11	6
Attu-2	52°48′42.2136″ N	173°18′1.8450″ E	1	-
Attu-3	52°48′42.2136″ N	173°18′4.0212″ E	4	-
Attu-4	52°49′45.7176″ N	173°17′25.1916″ E	12	11
Attu-5	52°49′56.5098″ N	173°18′42.3984″ E	4	3
Attu-6	52°50′22.7256″ N	173°15′44.0856″ E	4	2
Attu-7	52°50′45.9306″ N	173°15′5.9358″ E	3	2
Attu-8	52°48′19.0326″ N	173°9′56.0124″ E	18	10
Attu-9	52°48′21.4704″ N	173°9′56.8830″ E	15	14
Attu-10	52°48′38.5236″ N	173°9′37.2702″ E	4	4
Attu-11	52°48′44.9784″ N	173°9′30.0240″ E	1	-
Attu-12	52°48′10.4862″ N	173°10′4.8174″ E	10	10
Attu-13	52°48′15.9150″ N	173°10′22.9908″ E	10	8
Attu-14	52°48′15.7926″ N	173°10′23.6598″ E	8	7
Attu-15	52°48′59.7888″ N	173°9′26.7372″ E	2	2
Attu-17	52°48′51.1776″ N	173°9′35.1684″ E	1	-
Attu-18	52°48′9.9246″ N	173°10′12.9756″ E	7	5
Attu-19	52°47′51.9210″ N	173°10′17.8998″ E	4	-
Attu-20	52°47′47.1156″ N	173°10′15.2322″ E	6	5
Attu-21	52°48′21.9018″ N	173°9′34.7394″ E	1	-
Total			126	89
*Chrysanthemum arcticum* subsp. *polaré*				
Bluff A	58°46′16.5354″ N	−93°50′34.6878″ W	1	-
SP	58°45′52.3044″ N	−94°3′23.7024″ W	1	-
WHWPl	58°46′34.4316″ N	−93°49′32.5488″ W	1	-
WHWPo	58°46′34.4316″ N	−93°49′32.5488″ W	1	-
PB	58°45′59.0070″ N	−94°5′36.4632″ W	1	-
Go	58°46′12.1182″ N	−94°9′9.2730″ W	1	-
Bluff C	58°46′54.3852″ N	−93°52′5.4156″ W	1	-
Bluff D	58°45′56.7720″ N	−94°6′36.0720″ W	10	-
ItTrI	58°45′48.3510″ N	−93°53′46.4856″ W	1	-
GB	58°45′58.6980″ N	−93°55′24.0666″ W	1	-
CB	58°46′26.2474″ N	−94°10′19.5018″ W	1	-
RBeTrI	58°45′52.5780″ N	−94°0′44.1138″ W	1	-
RBw	58°45′47.0010″ N	−94°2′26.0664″ W	1	-
CMe	58°47′4.6782″ N	−94°11′33.5544″ W	2	-
Bluff CTrI	58°46′54.3852″ N	−93°52′5.4156″ W	3	-
Nome	N/A	N/A	18	17
Total			45	17
Grand Total			225	106

A total of 225 genotypes were sampled as follows: *n* = 54 *C. arcticum* from nine populations, *n* = 126 *C. a.* subsp. *arcticum* from 20 populations, and *n* = 45 *C. a.* subsp. *polaré* was collected from 16 populations (Table 1).

**Table 2:** Hand-pollinated crosses (family, female parent, and male parent) used to generate F_1_, segregating progeny where both parents and offspring had SNP genotype data used to examine heterozygosity, the number of pollinations (inflorescences), total seed set (number of seeds sown), and number (%) germinated seedlings used in the experiment.

Family	Female Parent	Male Parent	Number of Pollinations (Inflorescences)	Total Seed Set (Number of Seeds Sown)	Number (%) Germinated Seedlings
20Ca-69	Old Valdez 3–8	Kenai 1–31	1	13 (13)	3 (23.1)
20Ca-71	Anchor Point 1–20	Kenai 1–31	1	100 (47)	33 (70.2)
20Ca-72	Old Valdez 3–8	Kenai 1–24	1	22 (22)	10 (45.5)
20Ca-73	Old Valdez 3–8	Anchor Point 1–28	1	417 (417)	372 (89.2)
20Ca-75	Kenai 1–24	Old Valdez 3–8	1	176 (176)	137 (77.8)

**Table 3:** Heterozygosity observed values in 5 families of *C. arcticum*: number of class I markers and percent heterozygosity, number of class II markers and percent heterozygosity, and Chi-squared values for diploid, triploid, and tetraploid ploidy levels. See text for delineation of test ratios, classes, Chi-square, and degree of freedom calculations.

Family (see Table 2)	Number of Offspring	Number of Homozygote by Heterozygote Markers (Class I)	% Heterozygosity (Class I Markers)	Number of Double Heterozygote Markers (Class II)	% Heterozygosity (Class II Markers)	Chi-Squared Value Diploid (df = 4)	Chi-Squared Value Triploid (df = 4)	Chi-Squared Value Tetraploid (df = 4)
20Ca-69	2	154	52%	599	74%	551 ***	358 ***	890 ***
20Ca-72	7	180	52%	787	75%	1283 ***	897 ***	2628 ***
20Ca-73	74	176	47%	783	71%	12,395 ***	15,384 ***	41,088 ***
20Ca-75	88	158	50%	854	72%	18,352 ***	18,846 ***	52,700 ***
20Ca-71	23	146	55%	926	75%	5768 ***	3298 ***	9866 ***

*** *p* ≤ 0.05.

## Data Availability

The datasets presented in this article are not readily available because of restrictions in the plant collection permits. Requests to access the datasets should be directed to the corresponding author.

## Abbreviations

The following abbreviations are used in this manuscript:

CLF	Constant liquid feed
HPS-HID	High-pressure sodium high intensity discharge
HR	Hour
K	Potassium
N	Nitrogen
P	Phosphorus
PSC	Pseudo-self-compatibility
SI	Self-incompatibility
